# Blood Pumps for Extracorporeal Membrane Oxygenation: Platelet Activation During Different Operating Conditions

**DOI:** 10.1097/MAT.0000000000001493

**Published:** 2021-06-01

**Authors:** Francesco Fiusco, Lars Mikael Broman, Lisa Prahl Wittberg

**Affiliations:** From the * Department of Engineering Mechanics, KTH, Stockholm, Sweden; † FLOW & BioMEx Center, KTH, Stockholm, Sweden; ‡ ECMO Centre Karolinska, Astrid Lindgren Children’s Hospital, Karolinska University Hospital, Stockholm, Sweden; § Department of Physiology and Pharmacology, Karolinska Institutet, Stockholm, Sweden.

**Keywords:** ECMO, centrifugal pump, thrombogenicity, low flow, computational fluid dynamics, platelet activation, backflow

## Abstract

Extracorporeal membrane oxygenation (ECMO) is a therapy used in severe cardiopulmonary failure. Blood is pumped through an artificial circuit exposing it to nonphysiologic conditions, which promote platelet activation and coagulation. Centrifugal pumps used at lower flow rates than their design point may lose pump efficiency and increase the risk of hemolysis. In this study, thrombogenic properties of two ECMO pumps designed for adult and neonatal use were evaluated using simulations in different flow scenarios. Three scenarios, adult pump in *adult mode* (4 L/min), adult pump in *baby mode* (300 ml/min), and neonatal pump used in its design point (300 ml/min), were simulated using computational fluid dynamics. The flow was numerically seeded with platelets, whose activation state was computed considering the stress history that acted along their respective path lines. Statistical distributions of activation state and residence time were drawn. The results showed that using the adult pump in *baby mode* increased the fraction of platelets with higher activation state confirming that low-pump flow rate impacts thrombogenicity. The neonatal pump showed a backflow at the inlet, which carried platelets in a retrograde motion contributing to an increased thrombogenic potential compared with the *adult mode* scenario.

## Abstract 2

Supplemental Digital Content is available in the text.

Extracorporeal membrane oxygenation (ECMO) is a life-saving therapy used to treat patients with cardiac or pulmonary failure. Its use has become more common in recent years.^1^ The ECMO circuit is composed of a centrifugal pump; a membrane oxygenator; and cannulae for drainage and reinfusion of the patient’s blood, tubing, and connectors. These results in exposure of the pumped blood to nonphysiologic conditions and contact with artificial surfaces that trigger platelet activation and promote coagulation.^2–4^ Thus, ECMO requires rigorous protocols for anticoagulation. Most commonly, intravenous unfractionated heparin or, alternatively, direct thrombin inhibitors are used to balance the risk of bleeding against the risk of thrombosis, which are the two major contributors to complications associated with ECMO.^5^

It is widely accepted that the thrombogenic potential of the ECMO circuit is due to mechanical reasons that promotes initiation of biochemical cascades. *In vivo* characterization of thrombus formation has been explored by several groups. Fujiwara *et al.*^6^ observed hemolytic damage and thrombus formation in animal experiments during heparin administration, while Hastings *et al.*^7^ assessed thrombogenicity by analyzing various components of the ECMO circuit where the centrifugal pump, tubing, and connectors were found to be major contributors of clot formation. Concerning available computational fluid dynamics (CFD) approaches, it has been put forth that Large Eddy Simulations (LES) are preferred when investigating flow structures in blood pumps.^2,8^ Moreover, efforts have been made to model platelet activation to assess the thrombogenicity of both implanted and extracorporeal devices used for circulatory support. These models are based on shear stress or by measuring markers of platelet activation from high-shear stresses during short exposure times.^9–12^ Platelet activation data have then been used to develop power-law functions based on stress and exposure time. In the modeling framework, the degree of platelet activation has been expressed in terms of *Platelet Activity State* (PAS), a measure of platelet activation normalized to the maximum amount of thrombin generated by a fully activated platelet. PAS has, for example, been used in studies on the thrombogenicity of both implantable blood pumps and different components of the ECMO circuit.^3,13^

Even though a pump is designed for a specific flow rate range, ECMO pumps are used in a wide span of flows. Today, extracorporeal carbon dioxide removal (ECCO2R) is an example as recently published by Gross-Hardt *et al.*,^14^ who studied ECMO pumps in the lower flow range of 0.5–1.5 L/min. They reported increased internal recirculation and increased shear stresses and hemolysis. Centrifugal pumps designed for adults and children >6 kg (flow range 0.5–8 L/min) have been used in neonates 3–6 kg at flows of 0.3–0.5 L/min due to the superior pump head compared to the same brand’s pump designated patients <6 kg. Evaluation of pump performance when used outside the “normal” flow range is important to improve understanding and avoid patient harm and make way for future pump designs.

While the overall thromboembolic risk to the patient depends on the circuit (pump, membrane oxygenator, cannulas, connectors) and the patient physiology and anatomy, it is important to understand the effect that each part induces on the clinical outcome, thus assessing the individual contribution of each component to the activation potential of the ECMO system.

The current study aimed to characterize flow dynamics and platelet activation of two different ECMO pumps with focus on a neonatal application.

## Materials and Methods

**Figure 1.**
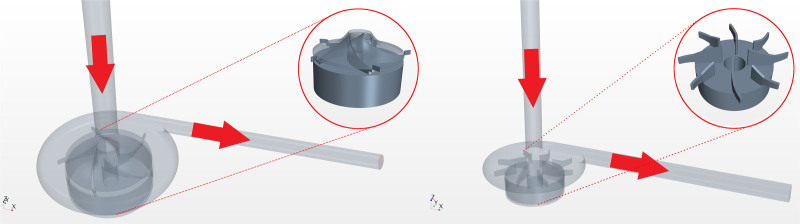
Pump geometries based on the PediVAS on the left and CentriMag on the right. The arrows indicate the direction of the flow.

This numerical study assesses the thrombogenic potential of two centrifugal pumps used in different simulated operational modes of ECMO. The two pumps considered were computer-aided design (CAD) generated geometries based on the design of the Levitronix CentriMag and PediVAS (PediMag on United States market) (Abbott, Chicago, IL), shown in Figure 1. The adult CentriMag pump has 3/8 inch in and outlets, a semi-open eight vane impeller with an outer diameter of 44 mm and a priming volume of 36 ml for a maximum operating pressure of 600 mm Hg. The PediVAS pump has ¼ inch in and outlets, a five-vane closed impeller, an outer diameter of 32 mm, and 16 ml priming; the maximum pressure is 540 mm Hg. The two pumps exhibit similar peak hydraulic efficiencies, but the PediVAS operates in a more efficient point in the lower flow rate range (0–3 L/min).^15^ The following three scenarios were simulated and investigated:

- Adult CentriMag pump as labeled: inlet flow rate of 4 L/min and 2,500 revolutions per minute (RPM), referred to in text as “adult mode.”
- CentriMag pump in off-label conditions: flow rate of 300 ml/min at 2,000 RPM, referred to as “baby mode.”
- PediVAS pump as labeled: flow rate of 300 ml/min at 3,000 RPM.

### Numerical Method

The flow field was fully resolved using the commercial CFD package Star-CCM+ (ver. 13.04.011). The numerical setup was according to the earlier description by Fuchs *et al*.^3^ However, in our study, the fluid was modeled as a Newtonian fluid mimicking blood with a density of 1,060 kg/m^3^ and dynamic viscosity of 0.003763 Pa s. To solve for the flow field, LES with a Wall Adapting Local Eddy-viscosity (WALE) sub-grid scale model was used. The timestep was fixed, using 1,440 time-steps for each full-pump revolution. At the inlet, a plug flow profile was applied (*i.e.*, a velocity perpendicular to the inlet surface with a magnitude corresponding to the prescribed flow rate), *no-slip* condition was imposed on all solid surfaces, and a prescribed pressure was applied at the outlet.

After the flow had reached statistical steady state, it was numerically seeded with 100,000 particles, *i.e., platelets*, that were followed using Lagrangian Particle Tracking (LPT). The trajectory of each particle was evaluated by


$$\frac{dX_{i}}{dt}=U_{i}\;\;\;\;\;\;\;\;\;\;m_{i}\frac{dU_{i}}{dt}=F_{i}$$ (1)

where *F*_*i*_ is the sum of all the forces acting on the particle, *X* the position, *U* the velocity, and *t* the time. The injected particles were characterized by a low Stokes number (2E-8), that is, they follow the flow without influencing it. The tracked PAS and residence time (time between injection at the inlet and absorption at the beginning of the outlet pipe) of each platelet were lumped into 40 bins. For each platelet, the shear stress history was recorded and used to compute PAS according to the model by Nobili *et al.*^9^ The model is cast in a dimensional power-law form based on a scalar norm of the shear stress tensor, accounting for history effects by integrating shear stresses along the platelet’s trajectory:


$$\frac{d\text{PAS}}{dt}=ca\tau {\;}^{b/a}D^{a-1}\;\;\;\;\;\;\;\;\;\frac{dD}{dt}=\tau {\;}^{b/a}$$ (2)

where the a, b, and c are empirically determined model parameters matched to *in vivo* activation measurements.^11^ D is the mechanical dose experienced by the platelet, and τ is the shear stress.

### Sensitivity Assessment

Regarding the flow field validation, the CentriMag pump was numerically verified through a mesh convergence study in the work by Berg *et al.*^2^ and Fuchs *et al.*^3^ Validation of the simulation setup of the PediVAS pump was carried out by (1) comparing the simulation results using water with experimental data and (2) a mesh convergence study using the Newtonian blood fluid model. The simulation showed good agreement with experimental data (discrepancy of 5.5%). The coarse, medium, and fine mesh had 4.5, 7, and 13 million computational cells, respectively. Convergence was assessed by considering the velocity profile on a vertical line in the gap region between magnet and pump housing, showing that differences between the medium and fine mesh were negligible, as shown in the additional material (Text, Supplemental Digital Content 1, http://links.lww.com/ASAIO/A666).

## Results

**Figure 2.**
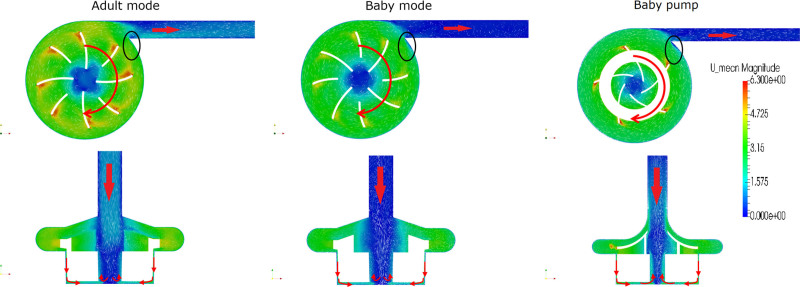
Phase averaged flow field of CentriMag adult mode (left), CentriMag baby mode (middle) and the PediVAS pump (right). The arrows represent flow direction and impeller rotation. The area circled in Black indicates the tongue of the pump.

The phase averaged flow fields of the three operating conditions are shown in Figure 2. There were clear differences between the three flow fields, especially regarding recirculation zones. For easy comprehension, animations are provided in the additional material showing top and front views of the velocity fields (Video, Supplemental Digital Content 2, 3, and 4, http://links.lww.com/ASAIO/A662, http://links.lww.com/ASAIO/A663, and http://links.lww.com/ASAIO/A664). Common for all three scenarios was the development of a recirculation region at the *tongue of the pump volute* (the sharp edge between the volute and outlet pipe, Figure 2). This recirculation region was markedly larger in the *PediVAS* case and associated with an increase in residence time. In the gap between pump housing and magnet, high-shear zones appeared in the form of azimuthally oriented vortices, so-called *Taylor-Couette* vortices.

The vortices at the tip of the blades were found to be more pronounced in the CentriMag *adult mode* and *PediVAS pump* scenarios as compared to the CentriMag *baby mode*, due to the lower inlet flow velocity and impeller rotation speed.

**Figure 3.**
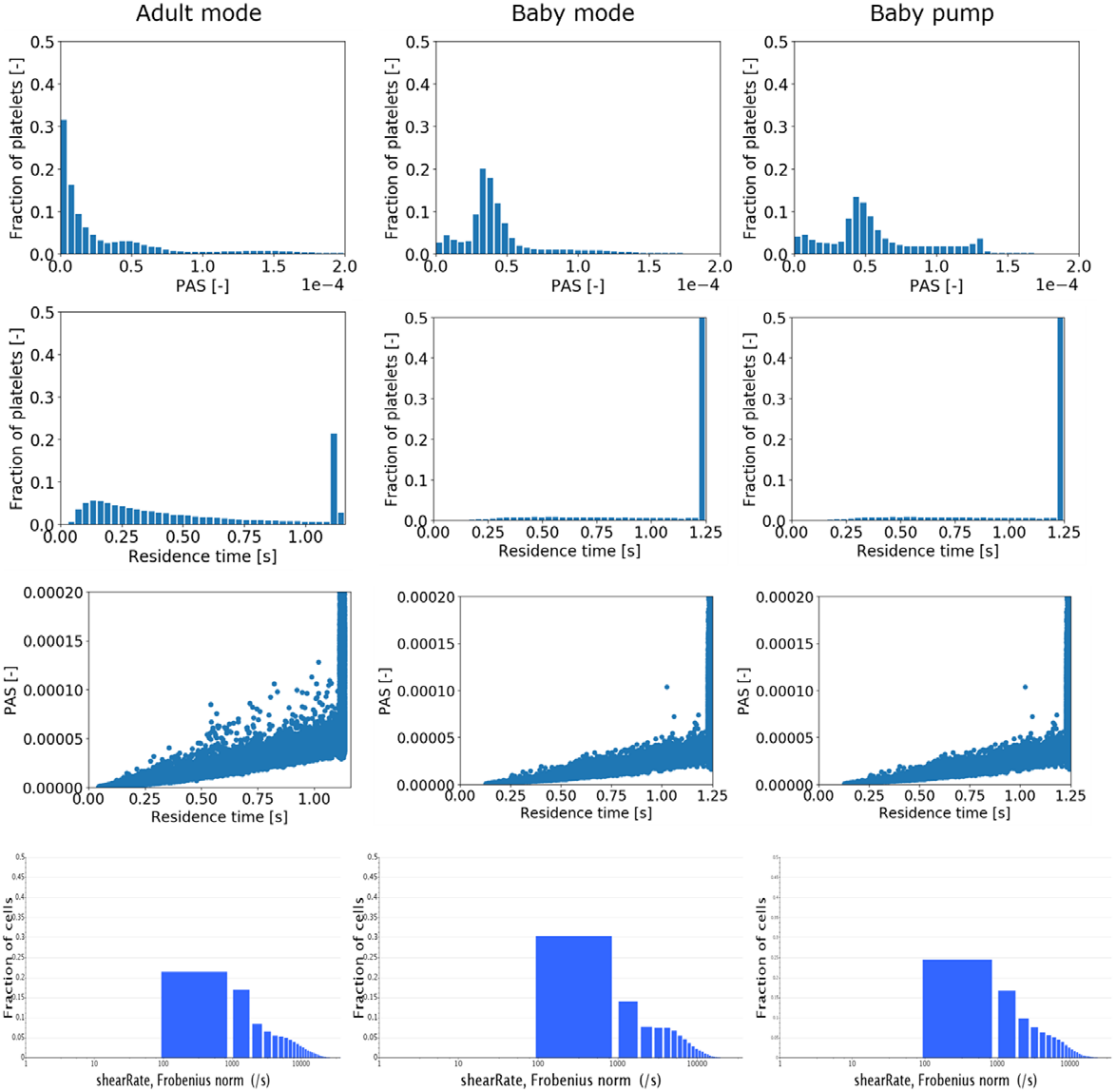
Distribution and of platelet activation state (PAS) and residence time of the CentriMag run in adult mode (3 L/min) and in Baby mode (300 ml/min) and the PediVAS run at 300 ml/min. The upper row shows fraction of platelets to PAS; the second row shows the fraction of platelets to residence time (the last bar is the platelet fraction still trapped / recycling in the pump after > 1.25 s), while the third panels show the impact of residence time on PAS for the respective pump/mode. The last row shows the shear rate distributions for the CentriMag run in adult mode (3 L/min) and in Baby mode (300 ml/min) and the PediVAS run at 300 ml/min. PAS, platelet activation state.

In all three scenarios, the distribution of shear rates across the domain was found to be quite similar, with the lowest shear rates starting at 100 s^–1^ and largest magnitudes <50,000 s^–1^, shown in the bottom panel of Figure 3.

### CentriMag Adult Mode

**Figure 4.**
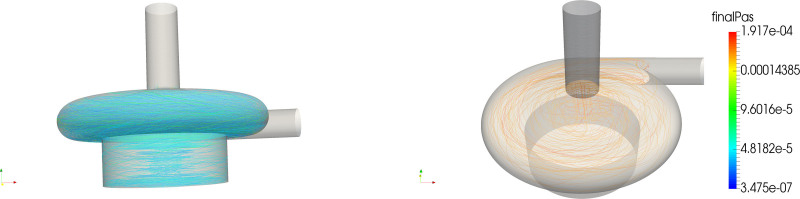
Path-lines for the CentriMag, adult mode of most activated platelets (left) and longest residing particles.

The distribution of platelets with respect to PAS and residence time is shown in the left panel of Figure 3. The correlation between residence time and PAS (Figure 3, third-row left) shows that, although the majority of the platelets with a higher PAS were the platelets that stayed longer in the pump volute, significant PAS was reached at shorter residence times as well. Figure 4 displays this phenomenon, as the most activated platelets (left) were platelets subjected to high-shear stresses in the volute, whereas the trajectories of the platelets that remain in the pump for a longer time traveled through the eye of the magnet and the gap between magnet and casing, getting trapped in the *Taylor-Couette* vortices.

### CentriMag Baby Mode

The flow field of the *baby mode* case (Figure 2, second panel) presented similar features with respect to the *adult mode*, albeit having substantially lower velocities. The recirculation zone at the tongue was also larger compared with the adult mode.

**Figure 5.**
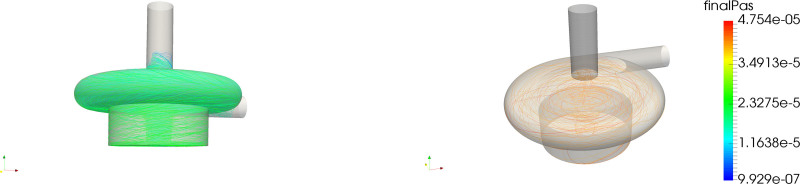
Path-lines for the CentriMag baby mode of longest residing (left) and most activated (right) platelets.

The PAS and residence time distributions (Figure 3, central panel) show that there were a larger fraction of platelets with higher degree of activation compared with the *adult mode*. The correlation plot (Figure 3, third-row mid) shows that the activation was mainly driven by residence time, also corroborated by the path-line trajectories shown in Figure 5 indicating that the most activated platelets were a subset of platelets with the longest residence times. The eye of the magnet promoted platelet activation to a higher extent compared to the *adult mode* case.

### PediVAS

**Figure 6.**
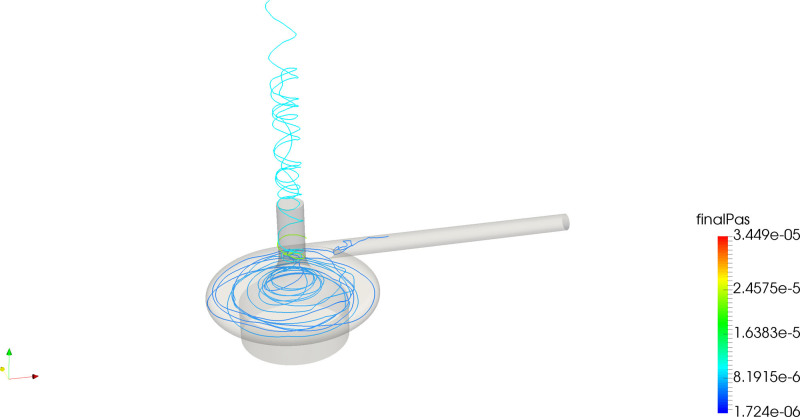
Path-lines of platelets for the PediVAS. At 300 ml/min, this unique finding shows that platelets were trapped and moved in retrograde direction to flow in the boundary layer.

**Figure 7.**
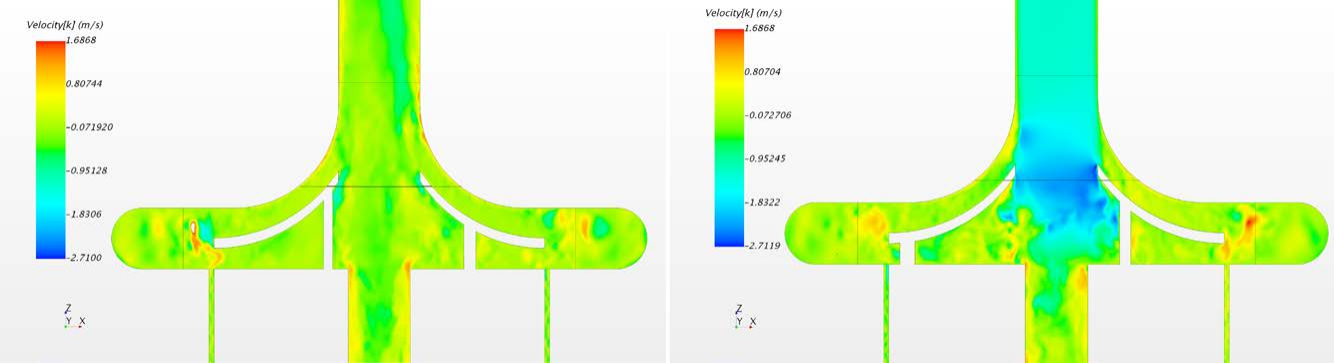
PediVAS in design conditions (300 ml/min, 3,000 RPM, left) and for a higher flow rate of 3 L/min, 3,000 RPM (outside labeled operating range, right). Color is function of vertical velocity. Notice the presence of flow structures at the wall.

**Figure 8.**
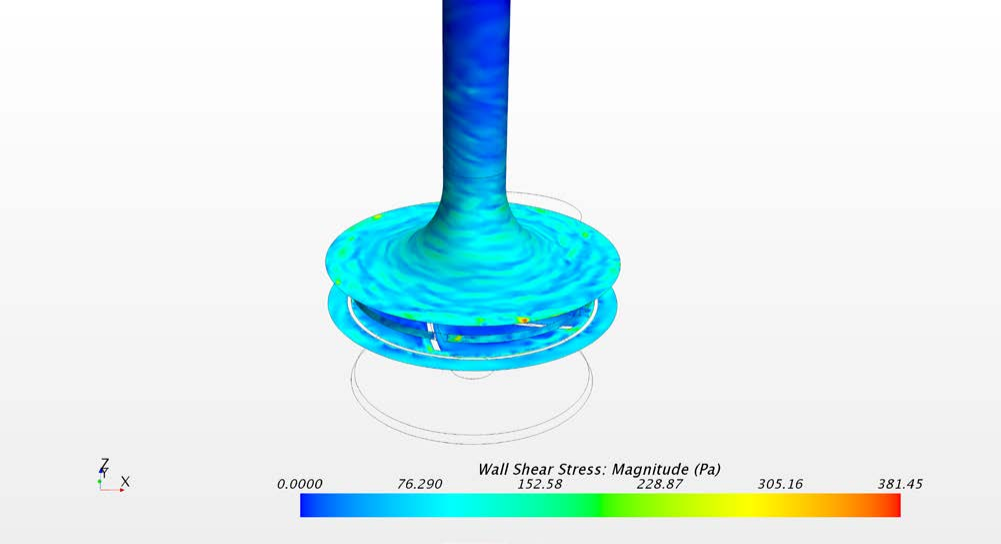
PediVAS: vortical structures in inlet pipe, represented with Wall shear stress.

**Figure 9.**
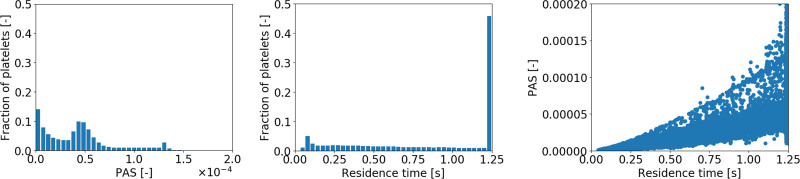
PediVAS: Histograms of repeated simulations without platelets in the boundary layer.

For the PediVAS, a distinct backflow developed where the inlet pipe joins the volute in the region referred to as the *shroud* (Video, Supplemental Digital Content 2, http://links.lww.com/ASAIO/A662). This backflow induced a backward swirling motion in the inlet pipe close to the tubing wall, depicted in Figure 6. To confirm this finding, additional simulations were carried out using both water and the Newtonian blood analog. This phenomenon was present with both fluids at 300 ml/min but also at 1,700 ml/min, which is the highest labeled flow rate for this pump. However, the phenomenon could not be observed when the pump was simulated with a flow of 3.4 L/min (Figure 7). This retrograde swirling motion (shown in Video, Supplemental Digital Content 5, http://links.lww.com/ASAIO/A665) carried platelets backwards toward the pump inlet and retrogradely out of the pump volute (Figure 6), confirmed by streaky flow structures appearing at the wall (Figure 8). Platelets experiencing this trajectory were subject to high shear for an extended amount of time that contributed to the PAS. This phenomenon also impacted the statistical distribution of PAS and residence time. Removing the platelets caught in the backward swirling motion at the tongue, the distributions in Figure 9 show a lower number of highly activated platelets.

**Figure 10.**
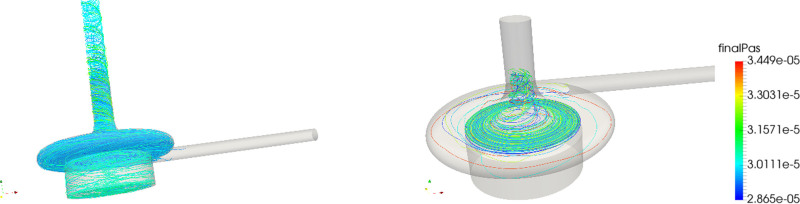
Path-lines for the PediVAS of longest residing (left) and most activated (right) platelets.

The distributions in terms of PAS and residence times are shown in the right panel column of Figure 3. The PAS distribution shows a similar shape as the CentriMag *baby mode*, with a slightly larger tail of more activated platelets. The correlation plot shows that most of the platelets with a higher PAS were the platelets that remained longer in the pump volute. As shown in the zoom-in of Figures 1 and 10, a critical region for both the increased residence time and platelet activation was the space between the roof (cover) over the PediVAS impeller and the volute. The right panel of Figure 10 depicts how the most activated platelets were trapped in the boundary layer above the cover, whereas the left panel illustrates the platelets having the longest residence times. As observed in both CentriMag scenarios, the gap between the magnet and housing, and the magnet eye were also critical regions. In the gap between pump housing and magnet, high-shear zones appeared in the form *Taylor-Couette* vortices.

## Discussion

Two ECMO pumps were investigated in different operating conditions with focus on flows used in the newly born. The adult pump labeled for patient weight >6 kg was tested as labeled but also at flows used in neonates. A neonatal pump was investigated at a similar labeled flow.

The first observation is that low pump flow rates seem to be *equally traumatizing to the blood* as high flow rates. The adult CentriMag pump tested in *baby mode* promoted PAS of a higher degree compared with operation in design condition. Similar effects at low flow rates have been observed for three other pump geometries regarding hemolytic properties,^14^ suggesting a link between hydraulic efficiency and thrombo-hemolytic properties.

The flow field of the *adult mode* was similar to what was already reported by Berg *et al.*^2^ and showed comparable behavior to the case presented by Fuchs *et al.*,^3^ but with higher PAS level due to the larger viscosity of the Newtonian blood analog used in the current simulations. The shear rate magnitudes confirm the validity of the Newtonian approximation used throughout the simulations. The difference in PAS suggests that simulations using water may underestimate the activation properties of ECMO circuit components. Relatively high levels of PAS were also reached at lower residence times, suggesting that activation mechanisms in this case were linked to high-shear stress magnitude, even if for a shorter time.

The CentriMag *baby mode* scenario showed different activation mechanisms as compared to the *adult mode* scenario, with the eye of the magnet becoming an important promotor of platelet activation. This is due to that platelets entering this region spend a comparatively longer fraction of time here since the vertical velocity is very low in the strong swirling motion shown in Figure 5. The PAS in this case was found to correlate to residence time rather than the level of shear, also supported by the fact that lower shear rates were recorded compared with the *adult mode* (especially in the wake of the blades, as shown in Figure 2), which lead to platelets being exposed to a lower shear for a longer time in regions such the aforementioned eye of the magnet. In spite of lower shear, these forces act on the platelets during a prolonged residence time, and hence the PAS may even be increased.

Regarding the PediVAS, the following findings should be pointed out:

1. For design conditions (300 ml/min), the PediVAS showed the longest residence time due to the backflow observed at the shroud. This finding is similar to flow structures that develop in compressors in *surge*, an undesired condition that occurs when a compressor is used for flow rates lower than designed for The fact that this phenomenon is also present in water simulations suggests this behavior to correlate with inlet conditions and can be observed throughout the pump label flow range. Based on our results, undesired backflow is vanishing at a higher flow rate (between 1,700 ml/min and 3,400 ml/min). The strong swirling motion transporting platelets backwards in the PediVAS was not observed in the other two pump configurations. Its presence can be compatible with experimental findings by Hastings *et al.*^7^ reporting observation of clots near the shaft of ECMO pumps in a large number of cases. However, experimental verification of this motion should be carried out, ideally in a clinical setting.
2. A major activation site was the gap between the cover of the closed impeller and the ceiling of the pump volute, trapping platelets in the boundary layer developed on the cover’s surface.
3. After elimination of the platelets captured by the backflow, the PAS for the PediVAS was still similar to that of the CentriMag *baby mode*. This suggests that the PediVAS design may be suboptimal for its intended target patient population due to both the backflow and the presence of cover on the impeller. A retrospective clinical study comparing CentriMag *baby mode* and PediVAS used in neonates would be beneficial to allow for a comparison of the activation properties during the two operating conditions.

The CentriMag *baby mode* and PediVAS scenarios showed comparable thrombogenic potentials, even though the latter was tested in its nominal flow range. For the PediVAS, the combined effect of covered impeller design and the inlet backflow contributed to an increased total PAS, both in terms of residence time and shear stresses. It is also worth highlighting that only the *adult mode* case reached washout in the considered simulation time.

Our results highlight the importance of further investigations on ECMO pumps during operating conditions and assessing the clinical outcome to further improve patient safety, as the observed flow structures are undesirable.

Some centers use a *bridge* in the circuit to shunt blood between the return and drainage tubing. Such shunt allows for a higher pump flow than the flow provided to the patient and is mostly used in the weaning phase of the neonate. However, it may thus be used to keep the pump flow within labeled range and circumvent the topic of increased PAS. Clinical practice has taught us that the bridge cause clotting problems when clamped (*i.e.*, stagnated flow) but also when open to flow, additional Y-piece connectors and flow regulation devices contribute to platelet activation.^3^

Moreover, these pumps are also used as ventricular assist devices where their thrombogenic potential plays a primary role in the overall incidence of thromboembolic complications.

### Limitations

These simulations were carried out considering blood as a Newtonian fluid, a valid assumption at shear rates >100 s^−1^.^16^ In the flow fields modeled here, even the zones at lowest shear rates have magnitudes of more than 100 s^−1^ with <0.1% of the platelets exposed to shear rates <30 s^−1^ below which the viscosity behavior completely differs from the Newtonian hypothesis.^16^ This suggests that the backflow could be observed also if a non-Newtonian model for blood was used, although higher viscosities could play a role in the blood flow in the inlet pipe. The chosen activation model does not consider platelet sensitization and its model parameters are calibrated for a shear rate range that is only a subset of the variability found in the presented scenarios.^17^ Nevertheless, the results are useful in a comparative perspective to characterize the behavior of the pumps during different operating conditions since the contribution to the activation state induced by the pump is assessed.

This work focuses mainly on the thrombogenic potential of the pumps with respect to platelet activation. Thus, other pathways of coagulation that may influence clinical physiologic events have not been considered in the modeling. However, platelet activation is involved in other pathways, for example, to the fibrinolytic and inflammatory cascades, thus giving an indirect insight into the influence on these processes.^18,19^

The use of pumps outside of their labeled flow range comes with potential risks and should be exercised with care. While some of the issues described in this study could be mitigated with the use of a recirculation line, the observed flow structures are undesirable and prompt the need for improved design of pumps for neonatal applications.

## Acknowledgments

Dr. Niclas Berg (KTH Engineering Mechanics, FLOW Center, BioMEx, Stockholm) is thankfully acknowledged for producing the CAD model including computational mesh for the CentriMag and for providing the LPT code.

## Supplementary Material



## References

[1] Makdisi G Wang IW: Extra Corporeal Membrane Oxygenation (ECMO) review of a lifesaving technology. J Thorac Dis. 7: E166–E176, 2015 26380745 10.3978/j.issn.2072-1439.2015.07.17 PMC4522501

[2] Berg N Fuchs L Prahl Wittberg L: Flow characteristics and coherent structures in a centrifugal blood pump. Flow, Turbul Combust. 102: 469–483, 2019

[3] Fuchs G Berg N Broman LM Prahl Wittberg L: Flow-induced platelet activation in components of the extracorporeal membrane oxygenation circuit. Sci Rep. 8: 13985, 2018. 30228350 10.1038/s41598-018-32247-y PMC6143512

[4] Karagiannidis C Brodie D Strassmann S: Extracorporeal membrane oxygenation: evolving epidemiology and mortality. Intensive Care Med. 42: 889–896, 2016. 26942446 10.1007/s00134-016-4273-z

[5] Lehle K Philipp A Zeman F: Technical-Induced Hemolysis in Patients with Respiratory Failure Supported with Veno-Venous ECMO - Prevalence and Risk Factors. PLoS One. 10: e0143527, 2015. 26606144 10.1371/journal.pone.0143527 PMC4659553

[6] Fujiwara T Nagaoka E Watanabe T: New generation extracorporeal membrane oxygenation with MedTech Mag-Lev, a single-use, magnetically levitated, centrifugal blood pump: preclinical evaluation in calves. Artif Organs. 37: 447–456, 2013. 23489176 10.1111/aor.12006

[7] Hastings SM Ku DN Wagoner S Maher KO Deshpande S: Sources of circuit thrombosis in pediatric extracorporeal membrane oxygenation. ASAIO J. 63: 86–92, 2017. 27660905 10.1097/MAT.0000000000000444

[8] Fraser KH Taskin ME Griffith BP Wu ZJ: The use of computational fluid dynamics in the development of ventricular assist devices. Med Eng Phys. 33: 263–280, 2011. 21075669 10.1016/j.medengphy.2010.10.014 PMC3053072

[9] Nobili M Sheriff J Morbiducci U Redaelli A Bluestein D: Platelet activation due to hemodynamic shear stresses: damage accumulation model and comparison to *in vitro* measurements. ASAIO J. 54: 64–72, 2008. 18204318 10.1097/MAT.0b013e31815d6898 PMC2756061

[10] Sheriff J Soares JS Xenos M Jesty J Slepian MJ Bluestein D: Evaluation of shear-induced platelet activation models under constant and dynamic shear stress loading conditions relevant to devices. Ann Biomed Eng. 41: 1279–1296, 2013. 23400312 10.1007/s10439-013-0758-x PMC3640664

[11] Soares JS Sheriff J Bluestein D: A novel mathematical model of activation and sensitization of platelets subjected to dynamic stress histories. Biomech Model Mechanobiol. 12: 1127–1141, 2013. 23359062 10.1007/s10237-013-0469-0 PMC3703483

[12] Ding J Chen Z Niu S: Quantification of shear-induced platelet activation : high shear stresses for short exposure time. Artif Organs. 2015: 39:576–583 25808300 10.1111/aor.12438

[13] Chiu WC Girdhar G Xenos M: Thromboresistance comparison of the HeartMate II ventricular assist device with the device thrombogenicity emulation- optimized HeartAssist 5 VAD. J Biomech Eng. 136: 021014, 2014. 24337144 10.1115/1.4026254 PMC4023653

[14] Gross-Hardt S Hesselmann F Arens J: Low-flow assessment of current ECMO/ECCO2R rotary blood pumps and the potential effect on hemocompatibility. Crit Care. 23: 348, 2019. 31694688 10.1186/s13054-019-2622-3 PMC6836552

[15] Dasse KA Gellman B Kameneva MV: Assessment of hydraulic performance and biocompatibility of a MagLev centrifugal pump system designed for pediatric cardiac or cardiopulmonary support. ASAIO J. 53: 771–777, 2007. 18043164 10.1097/MAT.0b013e31815dbf66 PMC3285494

[16] Cherry EM Eaton JK: Shear thinning effects on blood flow in straight and curved tubes. Phys Fluids. 25: 073104, 2013

[17] Fuchs G Berg N Broman LM Prahl Wittberg L: Modeling sensitivity and uncertainties in platelet activation models applied on centrifugal pumps for extracorporeal life support. Sci Rep. 9: 8809, 2019. 31217491 10.1038/s41598-019-45121-2 PMC6584555

[18] Margraf A Zarbock A: Platelets in inflammation and resolution. J Immunol. 203: 2357–2367, 2019. 31636134 10.4049/jimmunol.1900899

[19] Suzuki Y Sano H Mochizuki L Honkura N Urano T: Activated platelet-based inhibition of fibrinolysis via thrombin-activatable fibrinolysis inhibitor activation system. Blood Adv. 4: 5501–5511, 2020. 33166409 10.1182/bloodadvances.2020002923 PMC7656914

