# Supplementary material for: Blood Pumps for Extracorporeal Membrane Oxygenation: Platelet Activation During Different Operating Conditions
Source: ASAIO J. 2021 Jun 1;68(1):79–86. doi: 10.1097/MAT.0000000000001493 (PMC8700320; doi:10.1097/MAT.0000000000001493)
Supplement: Supplementary file 5 [file mat-68-79-s005.pdf]

Meshing details

The mesh was composed of polyhedral cells with a target size of 0.0003 m in the bulk of the flow. A refinement was applied in the rotating region to reproduce a structured-like mesh setup, shown below.

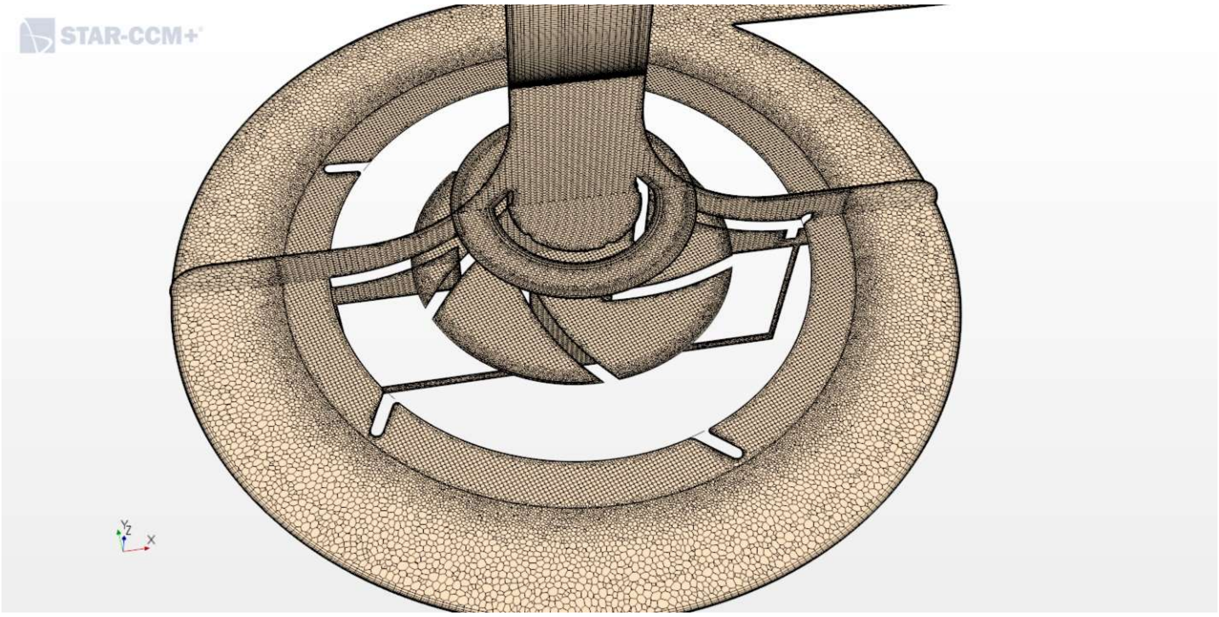

Figure 1 Mesh of the PediVAS pump

In the static region seven prism layers were used at the walls, whereas three prism layers were employed in the rotating region due to the presence of small gaps that did not allow for more layers. A refinement was also applied on the interface between the static and rotating region to ease the transition and limit the discontinuities between the two regions. The incompressible Navier-Stokes equation were solved with a segregated solver with implicit time stepping (fixed timestep so that a pump revolution corresponds to 1440 timesteps), using a LES approach with 7 inner iterations per timestep and a WALE subgrid scale model. To prove mesh convergence, the velocity profiles over two lines for the coarse (4.5 million cells), medium (7 million cells) and fine (13 million cells)

mesh were plotted. The locations of the lines are in the Taylor-Couette and gap region respectively and are shown below.

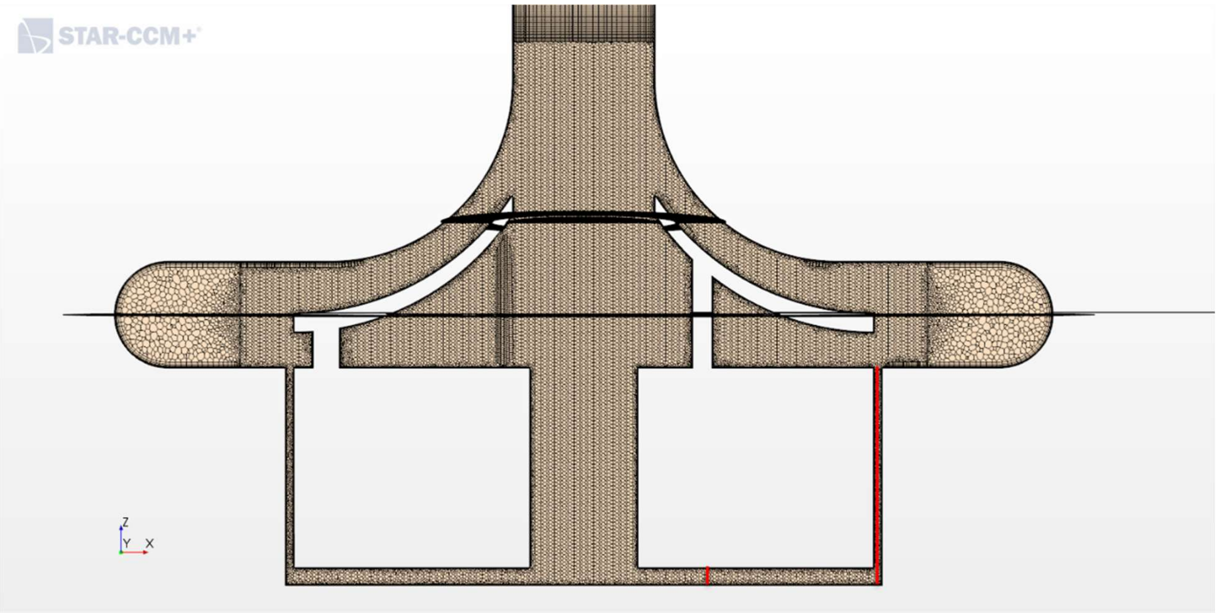

Figure 2 Locations of the velocity lines

The velocity profiles on those lines are shown in the following Figure.

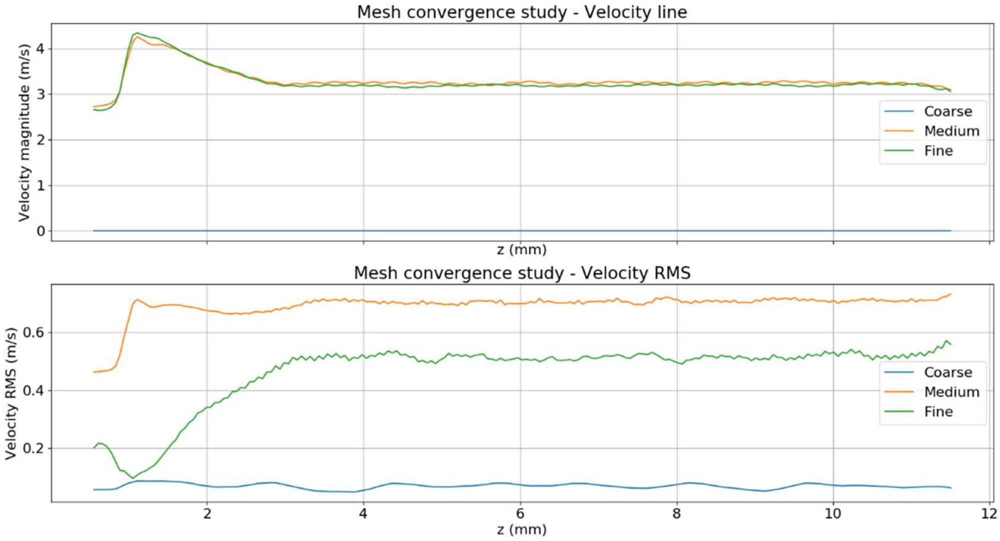

Figure 3 Velocity profiles in the Taylor-Couette region

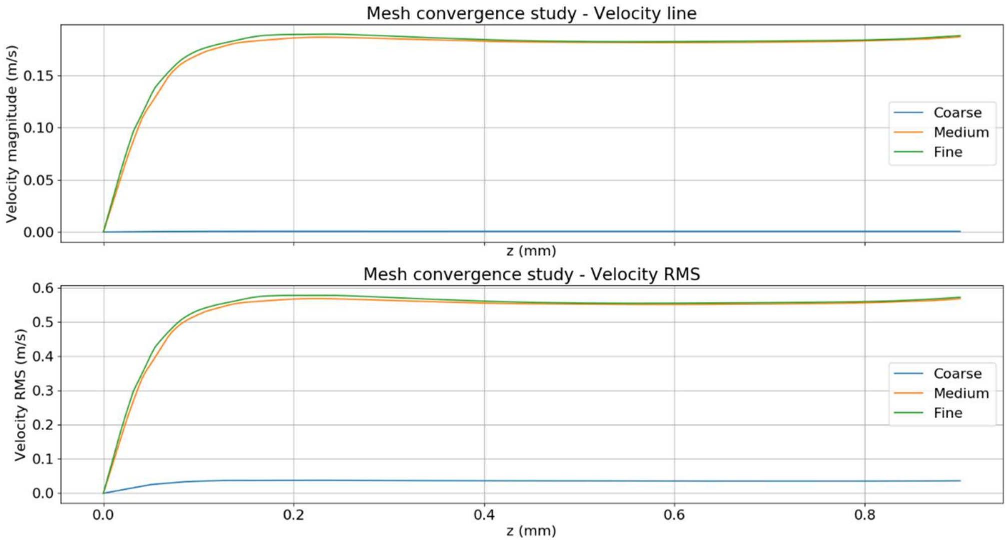

Figure 4 Velocity profiles in the gap region
